# Public Interest in Psilocybin and Psychedelic Therapy in the Context of the COVID-19 Pandemic: Google Trends Analysis

**DOI:** 10.2196/43850

**Published:** 2023-12-29

**Authors:** George Danias, Jacob Appel

**Affiliations:** 1 Icahn School of Medicine at Mount Sinai New York, NY United States

**Keywords:** psilocybin, Google Trends, COVID-19, medical informatics, depression, anxiety, substance use, social media, trend analysis, antidepressant

## Abstract

**Background:**

Psychedelic substances have demonstrated promise in the treatment of depression, anxiety, and substance use disorders. Significant media coverage has been dedicated to psychedelic medicine, but it is unclear whether the public associates psilocybin with its potential therapeutic benefits. The COVID-19 pandemic led to an increase in depression, anxiety, and substance abuse in the general population.

**Objective:**

This study attempts to link increases in interest in these disorders with increases in interest in psilocybin using Google Trends.

**Methods:**

Weekly interest-over-time Google Trends data for 4 years, from the week of March 11, 2018, to the week of March 6, 2022, were obtained for the following terms: “psilocybin,” “psychedelic therapy,” “cannabis,” “cocaine,” “antidepressant,” “depression,” “anxiety,” and “addiction.” Important psilocybin-related news and the declaration of the pandemic were noted. Trends data for each of the queried terms were plotted, and multiple regression analysis was performed to determine the slope of the prepandemic and postpandemic data with 95% CIs. Nonparametric Tau-*U* analysis was performed correcting for baseline trends. Results from this test were used to make inferences about the pre- and postpandemic trends and inferences about the change in overall level of searches between the 2 groups.

**Results:**

Tau values for prepandemic data were significant for stable trends, all ranging –0.4 to 0.4. Tau values for postpandemic data showed positive trends for “psilocybin,” “psychedelic therapy,” and “antidepressant.” All other trends remained stable in the range of –0.4 to 0.4. When comparing Tau values for pre- and postpandemic data, overall increases in relative search volume (RSV) were seen for “psilocybin,” “psychedelic therapy,” and “anxiety,” and overall decreases in RSV were seen for “depression,” “addiction,” and “cocaine.” Overall RSVs for “cannabis” and “antidepressant” remained stable as Tau values ranged between –0.4 and 0.4. In the immediate aftermath of the declaration of the pandemic, drop-offs in interest were seen for all terms except for “anxiety” and “cannabis.” After the initial shock of a global pandemic, “psilocybin” and “psychedelic therapy” groups demonstrated increases in interest trends and overall RSV.

**Conclusions:**

These data suggest that overall interest in “psilocybin” and “psychedelic therapy” increased at higher rates and to higher levels after than before the declaration of the pandemic. This is consistent with our hypothesis that interest increased for these treatments after the pandemic as incidence of depression, anxiety, and addiction increased. However, there may be other drivers of interest for these topics, since interest in antidepressants—the typical pharmacologic treatments for depression and anxiety—followed the expected pattern of drop-off and accelerated interest back to prepandemic levels. Interest in “psilocybin” and “psychedelic therapy” may have also been partially driven by popular culture hype and novelty, explaining why interest increased at a higher rate post pandemic and continued to grow, surpassing prior interest.

## Introduction

Despite a tumultuous history of discovery, exploration, criminalization, politicization, and mysticism, psychedelic substances are slowly re-entering the public eye and are attracting curiosity from scientists and laypeople alike [1]. Interest in and acceptance of psychedelic substances for the treatment of psychiatric illness has increased significantly over the last 2 decades [2]. Most notably, Johns Hopkins Medical Center’s gaining federal approval for research of psychedelic substances in 2000 and their opening of the Center for Psychedelic Research in 2019 paved the way for the current psychedelic research era [3]. Psilocybin, the psychoactive substance in several species of mushrooms, is of particular interest due to promising findings in multiple studies [4]. Psilocybin has been shown to significantly improve symptoms in patients with depression [5] and anxiety [5] and reduce substance use in patients with addiction [6,7].

The COVID-19 pandemic has taken a large toll on the physical and mental health of the world’s population. Specifically in the United States, over 1 million people have died because of SARS-CoV-2 infection at the time of writing [8]. The United States has also experienced the highest rate of COVID-19 deaths per capita among all high-income nations, with a mortality of 3038 per 1 million population [9]. In the United States, rates of depression and anxiety have increased since the beginning of the pandemic [10], continuing an upward trend that has been steady for years [11]. Psychiatric hospitalizations and rates of suicidal ideation have also risen [11]. Additionally, substance use has increased significantly during the pandemic [12].

Given the toll that the COVID-19 pandemic has had on mental health in the realm of depression, anxiety, and substance use, and given that psilocybin has shown promise in treatment of these mental illnesses, we investigated whether public interest in psilocybin and psychedelic therapy has been affected by the COVID-19 pandemic. We used Google Trends data [13] to determine whether trends in searches for psilocybin and psychedelic therapy were different in the periods before and after the onset of the COVID-19 pandemic and whether any such effect is also present for conditions that are potential targets of such therapy.

Google Trends is a database and data visualization tool, which tracks the search terms entered in Google’s search engine—the largest search engine on the internet [14]. Because of its use of metadata, Google Trends is useful for tracking public interest in topics in real time, with minimal supervision bias [14]. Google Trends data have been used to correlate incidences of tick-borne diseases [15], vaccine hesitancy [16], and sarcopenia [17] to the interest in their search terms. Relevant to this study, Google Trends has also been used to analyze searches related to anxiety before and after the onset of the COVID-19 pandemic [18].

## Methods

### Overview

Weekly interest-over-time Google Trends data for 4 years, from the week of March 11, 2018, to the week of March 6, 2022, were downloaded from Google Trends [13] for the following terms:, “psilocybin” (topic: chemical compound), “cannabis” (topic: drug), “cocaine” (topic: drug), “psychedelic therapy” (topic), “antidepressant” (topic), “depression” (topic: mood), “anxiety” (topic: emotional disorder), and “addiction” (topic). The search was performed on August 23, 2022. The period studied was selected such that it extended 2 years prior to and 2 years after the date of the declaration of the COVID-19 pandemic by the World Health Organization (WHO), a commonly referenced point for the start of the pandemic in the United States.

Topic data include a group of terms related to the search query, including typographical errors and the same term in different languages. For example, the topic “psilocybin” would return searches for “psilocybin mushrooms” as well as “psilocibina” (Spanish for “psilocybin”). Data were only collected from searches in the United States. Data were downloaded in tabular format as relative search volumes (RSVs) for each week within the study period (a total of 208 weeks). RSVs are normalized to the highest absolute number of searches within the query period (in this case, 4 years) with the greatest number of searches having an RSV of 100. Each term was queried individually to avoid normalization to a different term.

To understand whether search “spikes” seen in the Google Trends data for the search term “psilocybin” were related to events that may have increased the public’s interest in psychedelic substances, a news media analysis was conducted using the New York Times [19] and Los Angeles Times [20] databases between March 11, 2018, and March 6, 2022. Search results were reviewed by investigators and events with the highest potential to impact Google Searches for psilocybin, such as popular media and election ballot measures, were documented and annotated on the trends data graph.

Data were graphed using Excel (Microsoft Corp) [21] and analyzed statistically using NCSS [22]. The slope and CIs for each of the queried terms for the pre- and postpandemic periods were calculated and compared using multiple regression. Tau-*U* analysis was performed correcting for baseline trends using the free web-based calculator from Single Case Research [23]. Results from this test were used to make inferences about pre- and postpandemic trends, and about the change in overall level of searches between the 2 periods.

### Ethical Considerations

The methods described above adhere to privacy and data protection regulations as well as the terms and conditions of Google’s search engine, which are available to all users. Google Trends data might not represent the entire population equally, potentially leading to biases; thus, conclusions drawn from these data must be considered with this in mind. Ethics review by our institution was not required, as these data are publicly available and do not include personal health information.

## Results

**Figure 1:**
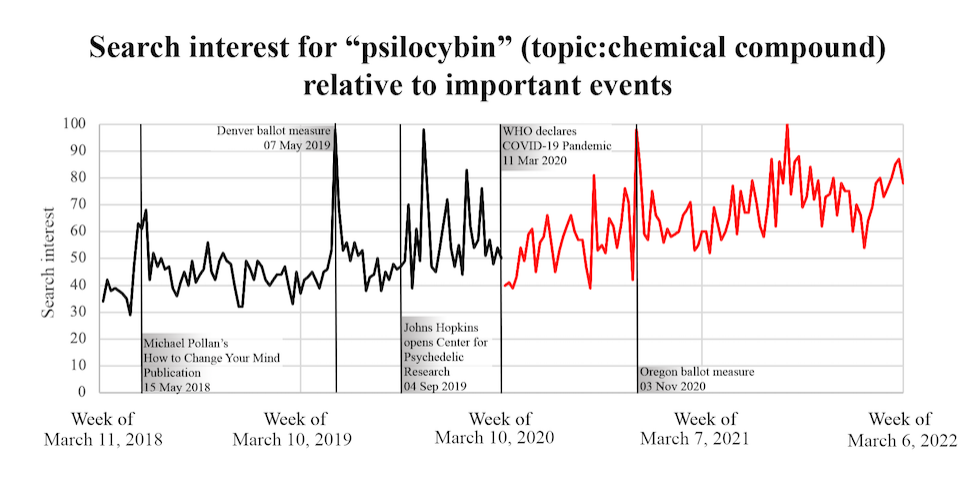
Google Trends search interest data for “psilocybin” (topic: chemical compound), with the World Health Organization’s declaration of the COVID-19 pandemic and newsworthy events related to psilocybin directly labeled on the chart. Data prior to the declaration of the COVID-19 pandemic are in black, while those after the declaration of the COVID-19 pandemic are in red.

RSVs for the term “psilocybin” (topic: chemical compound) during the study period are presented in Figure 1. Visual inspection suggests that although certain events (the publication of Michael Pollan’s psychedelic-related popular nonfiction book, the passing of the Denver ballot initiative, and the passing of the Oregon ballot initiative) appear to correlate with spikes in the relative number of searches for “psilocybin,” others, such as the opening of the Johns Hopkins Center for Psychedelic and Consciousness Research, did not.

**Figure 2:**
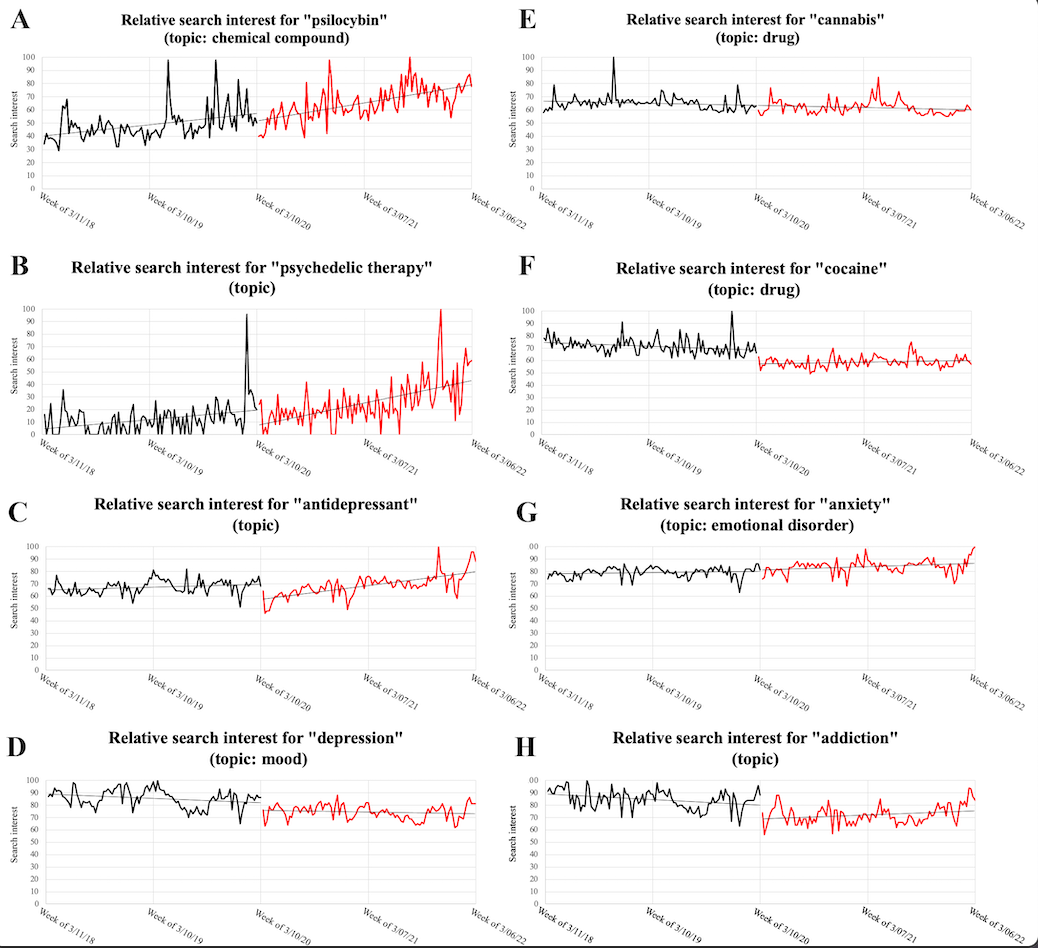
Google Trends search interest data for (A) “psilocybin” (topic: chemical compound), (B) “psychedelic therapy” (topic), (C) “antidepressant” (topic), (D) “depression” (topic: mood), (E) “cannabis” (topic: drug), (F) “cocaine” (topic: drug), (G) “anxiety” (topic: emotional disorder), and (H) “addiction” (topic). Lines of best fit are included. Data prior to the declaration of the COVID-19 pandemic are in black, while those after the declaration of the COVID-19 pandemic are in red.

Slopes (and 95% CIs) of the lines of best linear fit (Figure 2) for each search term over time for the periods before and after the declaration of the COVID-19 pandemic are listed in Table 1. Nonoverlapping CIs in the period before the declaration of the COVID-19 pandemic with those of the CIs after the declaration of the COVID-19 pandemic are highlighted. Positive trends were observed for the terms “psilocybin,” “psychedelic therapy,” and “antidepressants” (searches increasing over time) before the declaration of the pandemic, no significant trends were observed for the terms “anxiety” and “cannabis,” and negative trends were observed for the terms “depression,” “addiction,” and “cocaine” (searched decreasing over time). After the declaration of the pandemic, positive trends were observed for the terms “psilocybin,” “psychedelic therapy,” “antidepressants,” “anxiety,” and “addiction,” no significant trends were observed for the terms “depression” and “cocaine,” and a negative trend was observed for the term “cannabis.” Significant differences were observed in the slopes for the terms “psychedelic therapy,” “antidepressant,” “addiction,” and “cocaine,” while slopes appeared similar for the terms “psilocybin,” “depression,” “anxiety,” and “cannabis.” Trends for terms “psychedelic therapy,” “antidepressant,” “addiction,” and “cocaine” increased (ie, higher slope after the declaration of the COVID-19 pandemic).

**Table 1:** Slopes of the lines of best fit for each search term before and after the declaration of the COVID-19 pandemic with 95% CIs and *R*^2^ values. Nonoverlapping CIs between the period before the declaration of the COVID-19 pandemic and the period after the declaration of the COVID-19 pandemic are shaded.

Search term	Period before the declaration of the COVID-19 pandemic			Period after the declaration of the COVID-19 pandemic	
	Slope (95% CI)	*R* ^2^	Slope (95% CI)		*R* ^2^
Psilocybin	0.162 (0.092 to 0.233)	0.17	0.264 (0.200 to 0.328)		0.392
Psychedelic Therapy	0.143 (0.066 to 0.219)	0.118	0.341 (0.250 to 0.432)		0.35
Antidepressant	0.045 (0.011 to 0.08)	0.064	0.212 (0.169 to 0.255)		0.484
Depression	–0.067 (–0.113 to –0.021)	0.077	–0.029 (–0.065 to 0.008)		0.024
Anxiety	0.018 (–0.008 to 0.044)	0.018	0.052 (0.018 to 0.086)		0.081
Addiction	–0.085 (–0.132 to –0.037)	0.11	0.065 (0.018 to 0.111)		0.07
Cannabis	–0.032 (–0.067 to 0.003)	0.032	–0.033 (–0.065 to –0.001)		0.038
Cocaine	–0.059 (–0.099 to –0.019)	0.076	0.027 (–0.002 to 0.055)		0.033

Because time-series data are often autocorrelated, we performed Tau-*U* analysis. Tau-*U* analysis is performed on ranks and is thus not affected by autocorrelation [24]. Tau-*U* values are reported in Table 2. For the period prior to the declaration of the COVID-19 pandemic, Tau-*U* values ranged from –0.271 to 0.323 but were statistically significant. Positive values, indicating a positive trend, were noted for the terms “psilocybin,” “psychedelic therapy,” “antidepressants,” and “anxiety,” while negative values, indicating a negative trend, were noted for the terms “depression,” “addiction,” “cannabis,” and “cocaine.” For the period after the declaration of the COVID-19 pandemic, Tau-*U* values were statistically significant (indicating a significant trend) for all terms except for “depression” and “cocaine.” Positive trends were noted for the terms “psilocybin,” “psychedelic therapy,” “antidepressants,” “anxiety,” and “addiction,” while negative trends were noted for “cannabis.” All trends had Tau-*U* values ranging –0.4 to 0.4, except for the terms “psilocybin,” “psychedelic therapy,” and “antidepressant,” which had positive trends outside of this range. After correcting for baseline trends, Tau-*U* values for the comparison of the overall level of searches in the period before the declaration of the COVID-19 pandemic with that during the period after the declaration of the COVID-19 pandemic were statistically different in a positive direction (indicating an overall increase in RSVs during the post–pandemic declaration period) for the terms “psilocybin,” “psychedelic therapy,” and “anxiety,” while they were statistically different in a negative direction (indicating an overall decrease in RSVs during the post–pandemic declaration period) for terms “depression,” “addiction,” “cannabis,” and “cocaine.” The overall level of searches for the term “antidepressant” was not statistically different between the pre– and post–COVID-19 pandemic declaration periods. As can be visually ascertained in Figure 2C, this was caused by a reduction in the RSVs for this term immediately after the declaration of the COVID-19 pandemic, which was offset by a significant increase in the trend of searches in the post–COVID-19 pandemic declaration period.

**Table 2:** Tau-U values for trends for each search terms during the periods before and after the declaration of the COVID-19 pandemic and for the overall level of searches during these periods. Post–COVID-19 pandemic declaration period trends were baseline corrected.

Search term	Pre–COVID-19 pandemic declaration period, Tau-*U* (*P* value)	Post–COVID-19 pandemic declaration period, Tau-*U* (*P* value)	Pre– vs Post–COVID-19 pandemic declaration periods, baseline-corrected Tau-*U* (*P* value)
Psilocybin	0.323 (*<.001*^a^)	0.473 (*<.001*)	0.541 (*<.001*)
Psychedelic Therapy	0.229 (*.001*)	0.415 (*<.001*)	0.42 (*<.001*)
Antidepressant	0.204 (*.002*)	0.538 (*<.001*)	0.003 (*.*97)
Depression	–0.168 (*.01*)	–0.119 (.07)	–0.672 (*<.001*)
Anxiety	0.131 (*.048*)	0.19 (*.004*)	0.514 (*<.001*)
Addiction	–0.205 (*.002*)	0.154 (*.02*)	–0.646 (*<.001*)
Cannabis	–0.146 (*.03*)	–0.153 (*.02*)	–0.354 (*<.001*)
Cocaine	–0.271 (*<.001*)	0.1 (.13)	–0.793 (*<.001*)

^a^Italicized values are statistically significant at *P*<.05.

## Discussion

### Principal Findings

In the aftermath of the COVID-19 pandemic, there have been increases in the prevalences of depression, anxiety, and substance use. Psilocybin has shown promise in the treatment of these mental illnesses, and we sought to investigate whether public interest in psilocybin and psychedelic therapy has been affected by the COVID-19 pandemic. We used Google Trends data to determine whether trends in searches for “psilocybin” and “psychedelic therapy” were different in the periods before and after the onset of the COVID-19 pandemic and whether any such effect is also present for conditions that are potential targets of such therapy. We additionally downloaded search trends for (1) psychiatric conditions for which psilocybin demonstrates preliminary therapeutic promise (depression, anxiety, and addiction), (2) a common medical treatment for depression and anxiety (antidepressant), (3) an illicit substance that has an associated use disorder and has known medical benefits (cannabis), and (4) an illicit substance that has an associated use disorder and has no known medical benefits (cocaine).

Google Trends data typically show sharp peaks and troughs as the number of searches fluctuate from week to week. To understand whether newsworthy events related to psilocybin were responsible for the peaks identified in the graph of the Google Trends data, we noted on the same graph the dates of some of these events as identified by a search of the New York Times and Los Angeles Times databases. As expected, there were significant peaks in the relative number of searches during the weeks of the passing of the Denver ballot initiative and the passing of the Oregon ballot initiative. Interestingly, but not entirely unexpected, given the high profile of the author of this psychedelic-related book, a notable peak appeared to coincide temporally with the publication of Michael Pollan’s popular, psychedelic-related, nonfiction book, *How to Change Your Mind*. Surprisingly, the opening of the Johns Hopkins Center for Psychedelic and Consciousness Research was not associated with a peak in psilocybin-related search traffic. This may be because of the relatively academic nature of this event, which the public did not engage with to the same degree as other events. The highest RSV for “psilocybin” occurred during the week of August 1, 2021. News analysis did not reveal any psilocybin-related events around this time. However, this spike in searches may have been related to social media posts that would not necessarily be captured in a news media analysis.

The declaration of the COVID-19 pandemic by the WHO was temporally associated with a drop in psilocybin-related searches. We did notice similar reductions for most of the search terms studied. Because of the momentous nature of this event and the extensive media coverage of COVID-19 at the time as well as the finite time for internet searches, it is likely that searches on the topic of the COVID-19 “crowded out” all other searches.

Interestingly, no visible drop-offs in the relative number of searches were seen for the terms “anxiety” and “cannabis.” A possible explanation for this finding may be that the news of a global pandemic triggered feelings of anxiety in a large part of the public, offsetting some of the expected reduction in searches caused by the “crowding out” phenomenon. Cannabis is a substance used commonly for its anxiolytic properties [25], so patterns of searches for “cannabis” are likely to mirror those for searches for “anxiety” in this context.

After the initial shock induced by the declaration of the COVID-19 pandemic, one would expect search data to revert to prepandemic levels and trends if the pandemic had no effect on those. However, for the terms “psilocybin” and “psychedelic therapy,” both the trends as well as the overall levels of searches increased in the post–COVID-19 pandemic declaration period. These findings are consistent with our hypothesis that interest in psychedelic therapy and associated pharmaceutical substances would increase given their potential role in the treatment of depression, anxiety, and addiction, and they have been reported to increase after the onset of the COVID-19 pandemic [10-12]. However, there may be other drivers of interest in these topics, since the overall relative number of searches in antidepressants—the typical pharmacologic treatment for depression and anxiety—was not significantly different between the periods before and after the declaration of the COVID-19 pandemic, despite a significant increase in the trend of the number of searches in the post–COVID-19 pandemic declaration period. This may be partially explained by the steep initial drop in searches for “antidepressants,” which the increase in the trend of post–COVID-19 interest could not compensate for in the 2 years of our analysis. It is possible that this positive trend could have continued and led to an overall higher level of post–COVID-19 interest in antidepressants, but this was beyond the scope of our analysis. Alternatively, this difference may be related to the fact that the topics “psilocybin” and “psychedelic therapy” likely account for far fewer searches than “antidepressants” and could thus be less resistant to a “crowding out” effect. Additionally, starting from a very low number of searches would potentially allow an increase in overall number of searches in the post–COVID-19 pandemic declaration era, driven by popular culture hype and novelty.

Despite an increase in addiction related to the COVID-19 pandemic, we did not observe a change in search trends for the term “cocaine”; this trend plateaued during both the period before as well as that after the declaration of the COVID-19 pandemic. In fact, the overall number of searches for this term declined in the period after the declaration of the pandemic. Although, in retrospect, this can be attributed to the fact that that cocaine use decreased during the years after the declaration of the pandemic [26], this information was not available to the authors at the time of data collection. Increases in addiction disorders in the post–COVID-19 era are more due to increases in opiate use, which has overcompensated for the reduction in addiction related to cocaine [27].

One of the interesting findings of this study is that the overall number of searches for the terms “depression” and “addiction” were lower in the post–COVID-19 pandemic declaration period than during the pre–COVID-19 pandemic declaration period. Trends were unchanged and essentially plateaued for the term “depression,” while they marginally increased for the term “addiction.” The prevalence of both disorders has been reported to have increased in the postpandemic era. We can speculate that the reduction in the overall number of searches for these 2 terms may have resulted from the fact that both conditions are known to decrease motivation and help-seeking behavior [28]. Thus, individuals experiencing these disorders may not have been motivated to search the web for help. Due to lockdowns, working from home, and decreased socializing in the aftermath of the pandemic, these disorders would also have been less evident to loved ones as well who may have initiated web-based searches. In support of this explanation, searches for the term “anxiety” did not follow the same pattern as those for “anxiety,” even when not reaching the level of a psychiatric disorder, can increase information seeking [29].

### Limitations

This study has several limitations. Due to the retrospective nature of Google Trends analysis, and the fact that it lacks specificity and context, it is not possible to determine whether the increase in search interests in “psilocybin” and “psychedelic therapy” was due to increases in anxiety, depression, and addiction disorders induced by the declaration of the pandemic or some other factor. For example, it is possible that individuals initiating the searches were just curious to learn what they were, rather than being interested in using psychedelics themselves. While media analysis only found a few news headlines related to psilocybin, which would capture the public’s attention, it is possible that psilocybin and psychedelics are mentioned more in the media in the bodies of articles or in social media, which would not be captured by headline screening. However, inclusion of other search terms in the analysis allows comparisons, which helps generate inferences.

While analysis was performed on many of the terms as “topics” to cover all possible ways that searchers might have keyed into the search bar, there is a chance that certain search terms were overlooked. For example, in reference to the search term “psilocybin,” “mushrooms” or “shrooms” could have been an initial search by a Google user with no awareness of the word “psilocybin,” even though they might refer to the same substance. Use of the “topics” feature of Google Trends, which includes a broader set of search terms related to the term directly being searched for, partially addresses this issue, although Google does not provide information about what specific terms are included together with the primary term.

Although there is some ambiguity surrounding the exact onset of the COVID-19 pandemic as the virus had been circulating before the disease was officially declared a pandemic by the Chinese Center for Disease Control and Prevention or the WHO, and different agencies have reported different start dates, it is reasonable to adopt the timing of the WHO's declaration of the pandemic as the point of reference in this study. For the purpose of this analysis, this timing provides a widely recognized reference point in the United States, which is where the data were gathered and coincide with a drastic increase in Google searches for the topic “COVID” (data not presented here but available on Google Trends) [13].

As with all studies using Google Trends data, this study relies on normalized values for search frequency. Although the normalization formula that Google uses is proprietary, it should not affect conclusions on trends and overall levels of searches in subsets of the data if the normalization has been applied uniformly within the total study period. Although an overall increase in internet searches in general may have increased during the work-from-home boom, we failed to detect drastic increases in all the terms queried. Finally, Google Trends data may not be representative of the entire population due to disparities in internet access and usage across different demographic groups. Despite these limitations, Google Trends data continue to offer valuable insights.

### Conclusions

The overall number of internet searches for “psilocybin” and “psychedelic therapy” was greater in the 2 years after the declaration of the pandemic than in the 2 years prior to that. This increase has resulted from an increase in the trend of searches in the post–pandemic declaration period and suggests an accelerated increase in interest in psilocybin and psychedelic therapy, possibly related to the medical applications of psychedelics in the wake of the pandemic because of documented increases in the prevalence of depression, anxiety, and addiction. Some widely reported events may have contributed to certain spikes in the number of searches for psychedelic terms during the study period.

Information gleaned from this analysis could help guide medical information, public service announcements, and product marketing. Association of psilocybin as a medical treatment in public consciousness could facilitate legislation, research, and treatment.

## Abbreviations

RSV: relative search volume
WHO: World Health Organization

## Data Availability

The data used in this study were sourced from publicly available data on Google Trends. Google Trends is a freely accessible web-based platform that provides insights into the popularity and search interest of various keywords and topics. As a public tool, Google Trends data offer researchers the opportunity to investigate and analyze trends in search behavior over time.

To access the data used in this study, interested researchers can visit the Google Trends website [13] and perform custom searches based on their specific research interests. The platform allows users to explore search volume patterns, regional interest, related queries, and other relevant information for a wide range of topics.

Due to the public nature of the data, no additional permissions or agreements are required for data access. However, it is important to note that while Google Trends provides valuable insights into search behavior, it does not disclose absolute search volumes or provide access to individual user data. The data obtained from Google Trends for this study do not contain any personally identifiable information and are anonymized and aggregated. To ensure transparency and reproducibility, the specific search terms and parameters used in this study are provided in the Methods section. Researchers interested in replicating or extending this study can use the same search terms and parameters to retrieve the data from Google Trends and perform their analyses.
